# The perspectives of adolescents conceived using surrogacy, egg or sperm donation

**DOI:** 10.1093/humrep/dey088

**Published:** 2018-04-12

**Authors:** S Zadeh, E C Ilioi, V Jadva, S Golombok

**Affiliations:** Centre for Family Research, University of Cambridge, Cambridge CB2 3RF, UK

**Keywords:** adolescence, donor conception, surrogacy, egg donation, sperm donation

## Abstract

**STUDY QUESTION:**

What are the perspectives of adolescents conceived using surrogacy, egg or sperm donation regarding their conception and the third party involved?

**SUMMARY ANSWER:**

The majority of adolescents described feeling indifferent about their conception, and yet simultaneously reported an interest in the third party involved, or were in contact with them.

**WHAT IS KNOWN ALREADY:**

There is an assumption that children conceived through reproductive donation will feel negatively about their origins in adolescence. However, little is known about the views of adolescents who have been conceived through different types of reproductive donation.

**STUDY DESIGN, SIZE, DURATION:**

Forty-four adolescents, all of whom had been told about their conception in childhood, participated in a semi-structured interview as part of the sixth phase of a longitudinal, multi-method, multi-informant study of assisted reproduction families in the UK.

**PARTICIPANTS/MATERIALS, SETTING, METHODS:**

All adolescents were aged 14 years, had been conceived using surrogacy (*n* = 22), egg donation (*n* = 13) or sperm donation (*n* = 9) to heterosexual couples, and varied in terms of their information about, and contact with, the third party involved in their conception. Semi-structured interviews were conducted in participants’ homes. Interviews were analysed qualitatively to determine adolescents’ perceptions of their conception, and their thoughts and feelings about the surrogate or donor involved.

**MAIN RESULTS AND THE ROLE OF CHANCE:**

Adolescents were found to feel positive (*n* = 7), indifferent (*n* = 32) or ambivalent (*n* = 5) about their conception. Amongst adolescents not in contact with the surrogate or donor, most were interested (*n* = 16) in the surrogate or donor, and others were ambivalent (*n* = 4), or not interested (*n* = 6) in them. Adolescents in contact with the surrogate or donor expressed positive (*n* = 14), ambivalent (*n* = 1) or negative (*n* = 1) feelings about them.

**LIMITATIONS, REASONS FOR CAUTION:**

Of 56 adolescents invited to take part in the study, 47 consented to take part, giving a response rate of 84%. It was not possible to obtain information from adolescents who do not know about their conception.

**WIDER IMPLICATIONS OF THE FINDINGS:**

The findings contradict the assumption that children conceived through reproductive donation will feel negatively about their origins in adolescence and suggest that it may be helpful to draw a distinction between adolescents’ feelings about their conception in general, and their feelings about the surrogate or donor in particular.

**STUDY FUNDING/COMPETING INTEREST(S):**

This study was funded by the Wellcome Trust [097857/Z/11/Z]. The authors have no conflicts of interest to declare.

## Introduction

Reproductive donation is a form of assisted reproduction in which a third party assists in the conception of a child, either through donated gametes (sperm or egg) or by hosting the pregnancy, either using their own egg (traditional surrogacy) or the commissioning mother’s (gestational surrogacy) (Richards *et al.*, 2012). The different genetic and gestational connections that children born through reproductive donation have with their parents have formed the basis of much psychological research into parent and child wellbeing and parent–child relationships, showing that families formed through reproductive donation generally do not differ from their non-assisted counterparts in terms of parents’ psychological wellbeing, children’s psychological wellbeing, and the quality of parent–child relationships. Yet, it is still assumed that children conceived using reproductive donation, and in particular, surrogacy, will feel negatively about their origins as they grow older (Golombok, 2015).

The body of empirical evidence on donor-conceived offspring’s perspectives is growing, but it is often limited to non-representative samples of adults conceived by sperm donation (Turner and Coyle, 2000; Hewitt, 2002; Cushing, 2010; Mahlstedt *et al.*, 2010; Blyth, 2012; Harrigan *et al.*, 2015). Studies of the views of donor-conceived children have tended to focus on children raised by single women (Zadeh *et al.*, 2017a, 2017b) or lesbian couples (Vanfraussen *et al.*, 2001, 2002, 2003; Tasker and Granville, 2011; Malmquist *et al.*, 2014; Van Parys *et al.*, 2016; Raes *et al.*, 2015). The only study to have sought the perspectives of donor-conceived and surrogacy children raised in heterosexual two-parent families is the UK Longitudinal Study of Assisted Reproduction Families (Blake *et al.*, 2010, 2014; Jadva *et al.*, 2012). Blake *et al*. (2010, 2014) found that at age 7, children understand little, but by age 10, most demonstrate at least a rudimentary understanding of their donor conception, and express either neutral or positive feelings about it. Similarly, children born through surrogacy generally feel positive about being born in this way at ages 7 and 10, and those in contact with the surrogate feel positive about these relationships (Jadva *et al.*, 2012). This article reports findings from the same longitudinal study when the children reached adolescence.

The transition from childhood to adolescence has been described as a crucial time for identity formation and the development of personal autonomy (Erikson, 1968; Steinberg and Morris, 2001; Smetana *et al.*, 2006; Tsai *et al.*, 2013). Given that this is also a time of increased understanding of biology and genetic relatedness (Richards, 2000; Williams and Smith, 2010), adolescence represents a unique developmental stage that may present particular challenges for those conceived through reproductive donation. A systematic review of the psychological adjustment of adolescents conceived through assisted reproduction found no differences between adolescents conceived through egg or sperm donation and those conceived naturally (Ilioi and Golombok, 2015). However, in most of the studies included in this review, fewer than 10% of children of heterosexual couples knew about their donor conception.

Recent research has shown that the rate of parental disclosure is increasing both amongst parents who have used donors who may be identified by offspring, and those who have used anonymous donors (Isaksson *et al.*, 2012; Salevaara *et al.*, 2013; Freeman *et al.*, 2016). However, heterosexual couples generally tell their children about their donor conception at a later stage than do single women and lesbian couples (Jadva *et al.*, 2009). Such later and/or accidental disclosure has been shown to relate to negative feelings about donor conception (Turner and Coyle, 2000; Hewitt, 2002; Jadva *et al.*, 2009; Blyth, 2012). The present longitudinal study found that families in which parents disclosed donor conception to their children before the age of 7 showed more positive mother–child relationships and higher levels of wellbeing at age 14, as rated independently by mothers and adolescents (Ilioi *et al.*, 2017).

Adolescents’ views of their donor conception have largely been obtained through research using questionnaire methods. These surveys have recruited adolescents via an online forum for those interested in making connections with the donor or those conceived using the same donor (Jadva *et al.*, 2009; Beeson *et al.*, 2011; Hertz *et al.*, 2013), or have focused on adolescents whose donors are willing to be known (Scheib *et al.*, 2005), or adolescents raised by two mothers (Bos and Gartrell, 2011) or single mothers (Slutsky *et al.*, 2016). Scheib *et al.*’s (2005) survey included a sample of six adolescents with identifiable donors in heterosexual two-parent families, finding that these adolescents were mostly comfortable with their conception, but were less likely to expect their parents to be positive about their request for the donor’s identity than adolescents raised by single women or lesbian couples. In Beeson *et al.*’s (2011) study of 759 donor-conceived offspring, 52.6% of whom were <18 years old, the 168 sperm donor-conceived offspring raised by heterosexual couples who answered the question about their current feelings about their conception reported feeling ‘indifferent’ (35.7%), ‘different’ (26.2%), ‘special’ (25.6%) or ‘confused’ (11.3%). However, the proportion of adolescent respondents was not reported.

This study is the first to have asked adolescents conceived through different types of reproductive donation (surrogacy, egg or sperm donation) directly for their views. It reports the thoughts and feelings of a systematic sample of adolescents who have been raised in heterosexual two-parent families that were initially recruited to the study when the adolescents were infants through fertility clinics, the UK Office for National Statistics, and Childlessness Overcome Through Surrogacy (COTS), the only UK surrogacy organization at the time. The study sought to ascertain whether or not children feel distressed about the circumstances of their conception or birth when they reach adolescence, and what they think and feel about the surrogate or donor involved.

## Materials and Methods

### Sample characteristics

The data analysed in this article are from the sixth phase of the UK Longitudinal Study of Assisted Reproduction Families that has examined the impact of reproductive donation on children’s psychological wellbeing and parent–child relationships from infancy to adolescence (Golombok *et al.*, 2017; Ilioi *et al.*, 2017) Mothers were the primary point of contact and had been asked for permission to be contacted for follow-up at the previous phases of the study. The mothers were telephoned when their child reached 14 years of age (see, Golombok *et al.*, 2004a, 2004b, for details of initial recruitment procedures).

Of the 56 adolescents who had been told about their conception by reproductive donation, 47 consented to take part, giving a response rate of 84%. Of those who took part, 44 adolescents were willing to discuss their conception (22 conceived through surrogacy (15 through traditional surrogacy, 7 through gestational surrogacy), 13 through egg donation, and 9 through sperm donation). The remaining three adolescents, who responded to the initial question of ‘Can you tell me more about how you were made?’ with ‘don’t know’, or otherwise diverted the interview to another topic, were asked no further questions, and their interviews were subsequently excluded from the analyses.

All participants came from different families, and all had been told about their conception in childhood. In total, 28 (64%) were females and 16 (36%) were males. All participants were born in the year 2000, before the removal of donor anonymity in the UK in 2005. Overall, 14 (64%) of the adolescents conceived through surrogacy had contact with their surrogate, and 2 (15%) of the adolescents conceived through egg donation had contact with their egg donor (see Table I for details about type of surrogate/donor and frequency of contact). None of the adolescents conceived by sperm donation knew their sperm donor.

**Table I:** Feelings about conception, the surrogate/donor, type of surrogate/donor and frequency of contact by method of conception.

	Sperm donation	Egg donation	Traditional surrogacy	Gestational surrogacy	Total
Feelings about conception					
Positive	0	1	4	2	7
Indifferent	7	10	11	4	32
Ambivalent	2	2	0	1	5
Total	9	13	15	7	44
Feelings about surrogate or donor for adolescents not in contact					
Interested	5	6	4	1	16
Ambivalent	1	3	0	0	4
Not interested	3	0	3	0	6
Total	9	9*	7	1	26
Feelings about surrogate or donor for adolescents in contact					
Positive		2	7	5	14
Ambivalent		0	0	1	1
Negative		0	1	0	1
Total		0	8	6	16
Type of surrogate/donor					
Unknown	9	11	7	1	28
Previously unknown^a^	0	0	4	1	5
Previously known^b^	0	2	4	5	11
Total	9	13	15	7	44
Frequency of contact amongst those in contact with surrogate/donor					
Weekly or more		0	0	3	3
Weekly to monthly		1	1	0	2
A few times a year		0	5	2	7
Less than once a year		1	2	1	4
Total		2	8	6	16

^*^Missing data (*n* = 2).

^a^Remained in contact after meeting for purposes of surrogacy.

^b^Family member/friend.

### Interview

All participants were administered a semi-structured interview on their own at home by a researcher trained in the study techniques. Participants were asked about their level of understanding, thoughts and feelings about their conception, their knowledge of, and feelings towards, their surrogate or donor, their questions for their surrogate or donor, their level of contact with their surrogate or donor, their discussions with their parents and other people about their conception and their surrogate or donor, and their thoughts and feelings about these discussions. Interviewers were trained to exercise caution in probing participants, to avoid distress. In the two cases in which it was deemed inappropriate to ask specific questions, data were recorded as missing. All interviews were audio-recorded and later transcribed without identifying information.

Written informed consent to participate was provided by all adolescents and their mothers. Participants were reminded that their responses would remain confidential, and that they could terminate the interview at any time, without giving a reason. Ethical approval was granted by the University of Cambridge Psychology Research Ethics Committee.

### Analysis

Data were analysed using a qualitative content approach (Schreier, 2014) that involved reading each transcript closely, producing data-driven categories that captured the content of the transcripts, and subsequently coding all participants’ responses according to these categories. After the initial analysis was complete, a second researcher trained in the approach reviewed and confirmed all categories and their content. The main categories are presented below, along with frequency counts, and illustrative quotations.

## Results

### How do adolescents feel about their conception?

Adolescents’ feelings were found to correspond to three distinct categories: positive (*n* = 7), indifferent (*n* = 32) and ambivalent (*n* = 5) (Table I).

#### Positive

Some adolescents expressed positive feelings about their conception, describing it as ‘cool’, ‘interesting’ and a ‘special’ fact about themselves:

‘I was really confused [when first told] but then afterwards I felt quite special.’ (Gestational surrogacy)

‘I felt really special [when first told] because no one else was like that… I think I still feel like that. It’s different but it doesn’t really make me different.’ (Gestational surrogacy)

Some of these adolescents also highlighted that their peers had responded positively:

‘Sometimes my friends ask me about it, they’re just like “oh, it’s really cool how you were made”…It’s a really cool process, and I’m always like, “oh, yeah, I was lucky I was made this way”.’ (Egg donation)

‘It’s cool, I think it’s cool [laughs]. I quite like talking about it because it’s an interesting fact about me… My friends thought it was cool as well [laughs].’ (Traditional surrogacy)

#### Indifferent

The vast majority of adolescents expressed feeling indifferent about their conception. Adolescents in this category had been conceived through each of the reproductive techniques:

‘I don’t think I really minded [when first told] to be honest…I still don’t really care. It doesn’t make any difference.’ (Sperm donation)

‘[I] don’t really mind. It doesn’t really affect my daily life.’ (Traditional surrogacy)

‘I don’t really mind. Yeah, I don’t really mind.’ (Egg donation)

‘I didn’t really care to be honest. I still don’t really care.’ (Gestational surrogacy)

Some adolescents who described feeling indifferent also stated that their conception did not change the nature of their relationship with their parents:

‘Um, it didn’t really bother me. Mum is still my mum. Dad is still my dad.’ (Traditional surrogacy)

‘I don’t think it really affects anything. I consider that my dad is still my dad, so.’ (Sperm donation)

Others explained that they were indifferent at the same time as being interested in the process, or that they were comfortable discussing it with others:

‘Um, [I’m] not like bothered about it but I think it’s quite interesting to know how I was made.’ (Egg donation)

‘I don’t get like emotional or anything, it’s just like talking about anything else.’ (Egg donation)

However, others described sometimes finding such social encounters difficult:

‘My friends ask questions that I don’t know the answer to or I don’t really want to know the answer to. Like “Where did your Mum get like the thing?”, “How much money did it cost?”, “How did you get like the sperm for it and stuff?”. I don’t really want to answer that.’ (Egg donation)

Surrogacy was described as particularly difficult to explain to peers:

‘When I was little, I used to get called fostered and it sort of got to me “I’m not fostered! You don’t understand because you’re dumb!”…Frustrating when they say like “Oh you’re adopted, you’re fostered…” (Traditional surrogacy)

#### Ambivalent

Some adolescents were ambivalent about their conception, and described a combination of different feelings:

‘It makes you feel like you weren’t a mistake…but I sort of feel like I’ve got that part missing.’ (Sperm donation)

‘Sometimes I can go, ‘Is it natural? Is it normal? Is it…a normal thing to happen?’ but kind of assure myself that it is fine.’ (Gestational surrogacy)

### How do adolescents not in contact with the surrogate or donor feel about them?

Most adolescents (*n* = 28) had no contact with their surrogate or donor. These adolescents were found to be interested (*n* = 16), ambivalent (*n* = 4) or not interested (*n* = 6) in the surrogate or donor (Table I).

#### Interested

The majority of adolescents expressed a desire to either know who the surrogate or donor was, or to meet them. A list of these adolescents’ responses to the question about what they would like to ask the surrogate or donor is provided in Table II. Within this group, there was much variation in the level of detail adolescents provided. Some adolescents described wanting to identify similarities between themselves and the surrogate or donor:

‘A lot of the time I think I have quite a lot of things in common with my mum, and then I think, “Oh, what about the things that I have in common with my donor?”.’ (Egg donation)

**Table II:** Questions adolescents who are interested in the surrogate or donor would like to ask them.

Question topic	Number of children who would like to ask about this	Examples
Reasons for surrogacy/donation	11	‘What made you consider donating?’ (SD) ‘What made you like want to donate to like a complete stranger?’ (ED) ‘Why did you decide to be a surrogate mother?’ (TS)
Interests	7	‘Do you get involved in any like, sport activities?’ (SD) ‘Do you have any animals?’ (ED)
Experience and feelings about surrogacy/donation	4	‘What was it like? How did you get approached about it? And how do you feel about it all?’ (ED) ‘How did it feel? Are you happy you did it?’ (TS)
Surrogate/donor’s family	4	‘If he has any children of his own’ (SD) ‘Are you married?’ (ED)
Circumstances at time of surrogacy/donation	3	*‘*Maybe how old was he when he donated’ (SD) ‘Did you know my mum at the time?’ (GS)
Knowledge or thoughts about family formed through surrogacy/donation	3	‘Have you ever given any thought to the people you donated eggs to?’ (ED) ‘Did you ever want to meet us?’ (ED)
Children conceived using same surrogate/donor	3	‘I’d ask her how many times she’s done it, the surrogacy’ (TS) ‘Have you met any other people who are your children?’ (SD)
Appearance	1	‘I would love to know what she looks like’ (ED)
Contact	1	*‘*Would you like to get in contact… would you like to keep in contact?’ (TS)
Background	1	‘Where are you from?’ (SD)
No questions	1	

SD = sperm donation; ED = egg donation; TS = traditional surrogacy; GD = gestational surrogacy.

While most adolescents said that they infrequently thought about the donor, a minority stated that they thought about their donor frequently, and increasingly since entering adolescence:

‘I would like to know who he is…quite a lot…Recently a lot more than I used to.’ (Sperm donation)

‘It’s more important to me now….Um, and I’m just always thinking about what she looks like.’ (Egg donation)

Others who wished to know the identity of, or to meet the surrogate or donor described them as a ‘real’ parent:

‘[I think about] who my real mum is…I just really want to know like, who she is and meet her maybe.’ (Traditional surrogacy)

‘Yeah probably like [to know] who he is, and maybe meet him because he is my dad, but I’m not too bothered.’ (Sperm donation)

Yet others expressed concern that their interest in the donor might impact upon their non-genetic parent:

‘I would like to know who he is…quite a lot. And I told my mum that and I don’t want to tell my dad that because I don’t know how he would feel about that, so…’ (Sperm donation)

‘I think mum might be a bit upset but she’d understand about it but…I’d only do it when I was 50 or 60…I don’t feel the need to now.’ (Egg donation)

One adolescent stated that they would like to meet the surrogate to express their gratitude:

‘Sometimes I think about it…wanting to meet my birth mum. [To] say thanks for being my birth mum [laughs]…It sounds really funny. Yeah, I don’t know, just thanks.’ (Gestational surrogacy)

#### Ambivalent

Four adolescents explained that they were either unsure, or expressed both wanting and not wanting to know more about, and/or to meet, the surrogate or donor:

‘I’ve gone so long without knowing about her, it’s just easier…I think I’d want to meet to see if I had anything in common.’ (Egg donation)

*‘*I heard it’s when you’re 18 or something you can try to find out who it was, so I would possibly consider doing that, but I don’t know really.’ (Egg donation)

#### Not interested

Those adolescents identified as ‘not interested’ (*n* = 6) in the surrogate or donor answered questions about whether they would like to know anything about, or to meet, the surrogate or donor with simply ‘no’, or ‘not really, no’.

### How do adolescents in contact with the surrogate or donor feel about them?

Those adolescents who had contact with their surrogate or egg donor (*n* = 16) were found to feel positive, ambivalent or negative about them (Table I). Most adolescents who had contact with the surrogate or egg donor explained that geographical distance or lack of time accounted for the (in)frequency of visits. Many adolescents also mentioned being in contact with the children and grandchildren of the surrogate or egg donor.

The large majority (*n* = 14) of adolescents described their relationship with the surrogate or egg donor positively. Many participants referred to them as a family friend, an aunt or a godparent, and reported having a close relationship:

‘[Our relationship is] a good one. But I don’t talk to her like she’s my mum and she doesn’t talk to me like I’m her daughter.’ (Traditional surrogacy)

‘She’s like family to me but we don’t see her that often because she lives quite far away.’ (Traditional surrogacy)

Other adolescents emphasized that they didn’t really know the surrogate or donor well enough to say much about them. One adolescent was ambivalent about the surrogate, and in another case, the relationship between the surrogate and the adolescent and their family had broken down, with negative feelings reported as a result.

## Discussion

This study sheds light on how adolescents make meaning of their conception during a developmental stage that is characterized by increased cognitive capabilities and marked identity development (Erikson, 1968; Steinberg and Morris, 2001). The majority of adolescents were indifferent about their conception, and were either interested in, or enjoyed positive relations with, the surrogate or donor. Not one of the adolescents was distressed about their conception or birth. These findings, obtained first-hand from a sample of donor-conceived and surrogacy children followed up from infancy to adolescence, suggest that the concern that children born through reproductive donation would be distressed about their origins in adolescence is unfounded, and that children who are informed when young of their conception through reproductive donation are accepting of this in adolescence.

Feelings of indifference towards their conception were found amongst adolescents conceived through each of the four types of reproductive donation under study: traditional surrogacy, gestational surrogacy, egg donation and sperm donation. Interestingly, adolescents’ lack of concern about their method of conception is consistent with their high levels of psychological wellbeing and the quality of relationships with their mothers at this age (Golombok *et al.*, 2017; Ilioi *et al.*, 2017). The finding that none of the adolescents described feeling negatively about their origins is perhaps explained by the fact that almost all of them had been told about their conception before the age of 7 (Ilioi *et al.*, 2017). It is also worth noting that some of the adolescents described feeling ambivalent about their conception, whereas others were particularly positive.

In describing their experiences, several adolescents referred to the actual or anticipated responses of others—namely, parents and peers—to their feelings. It is noteworthy that a minority of adolescents described feeling concerned that their interest in their conception might upset the parent to whom they have no genetic connection, a finding that echoes earlier research (Scheib *et al.*, 2005; Jadva *et al.*, 2009; Beeson *et al.*, 2011). With regards to their peers, as in research on donor-conceived children raised by single women and lesbian couples (Vanfraussen *et al.*, 2002; Raes *et al.*, 2015; Van Parys *et al.*, 2016; Zadeh *et al.*, 2017a), a minority of adolescents also described issues arising from peers’ lack of understanding of reproductive donation, and in particular, surrogacy.

In terms of adolescents’ thoughts and feelings about the surrogate or donor, as in previous research on donor-conceived samples (Vanfraussen *et al.*, 2003; Scheib *et al.*, 2005; Jadva *et al.*, 2009; Rodino *et al.*, 2011; Slutsky *et al.*, 2016; Persaud *et al.*, 2017), many of the adolescents who were not in contact with the surrogate or donor expressed an interest in them. In a previous study of adolescents conceived by donor insemination to single women and lesbian couples, mother–child relationship quality was found to impact upon adolescents’ curiosity about the donor, such that adolescents who were securely attached to their mothers were more interested in exploring their donor conception than were those who were insecurely attached (Slutsky *et al.*, 2016). Given that the present sample was found to show high quality mother–child relationships (Golombok *et al.*, 2017; Ilioi *et al.*, 2017), the adolescents’ interest in the surrogate or donor is to be expected.

Adolescents who were interested in the surrogate or donor mostly wanted to know more about why they had donated or acted as a surrogate, and some had questions about the surrogate or donor’s family, or children conceived using the same surrogate or donor. Previous studies of adolescents born through reproductive donation who use the internet to try to connect with donor relations have shown that adolescents desire to learn more about the donor and other children who share their genetic material in order to better understand themselves (Jadva *et al.*, 2009; Persaud *et al.*, 2017). The fact that similar results are found with the present sample of adolescents, recruited systematically to the study when they were aged 1, suggests that feelings of curiosity amongst donor-conceived adolescents are not simply an artefact of sampling donor-conceived offspring who are actively searching for their donor relations. Of equal significance is that some of the adolescents reported having no interest in knowing more about, or meeting, the surrogate or donor. The feelings of these adolescents have not been captured by such previous studies.

While most of the adolescents who had contact with their surrogate or donor continued to have positive relationships with them (Jadva *et al.*, 2012), one adolescent reported that their relationship with the surrogate was now negative. Coupled with the fact that a minority of adolescents reported an increase over time in the extent to which they were thinking about their unknown surrogate or donor, this finding attests to the importance of studying the thoughts, feelings, and experiences of donor-conceived and surrogacy children over time. Relatedly, it is worth considering that the adolescents in this study were all conceived prior to legislative changes in the UK, which have ensured that since 2005 those who are donor-conceived will be able to identify the donor once they reach the age of 18, in effect inadvertently creating potentially distinct experiences amongst donor-conceived individuals conceived prior to, and after, these changes. However, the adolescents in this study may or may not engage in searching behaviour in the future. Initial insights from a longitudinal study of children with identifiable sperm donors suggest that fewer offspring raised by heterosexual couples request their donor’s identity in early adulthood than do those raised by single women and lesbian couples (Scheib *et al.*, 2017).

The findings of this study are based on a sample of adolescents who are all aware of their conception and have been raised in heterosexual two-parent families, and therefore tell us little about the experiences of adolescents in other family circumstances, or those who remain unaware of the circumstances of their conception or birth. Indeed, many parents in the overall sample of this longitudinal study have not told their children about the circumstances of their conception (Ilioi *et al.*, 2017). Despite these limitations, the participants in this study are among the first donor-conceived and the first surrogacy adolescents to be interviewed about their thoughts and feelings on how they were conceived, as the rates of disclosure of donor conception have until recently been very low, and 84% of those who were approached agreed to participate.

Although there has been much concern about how children conceived using reproductive donation would feel about their origins as they grow older, the adolescents in this study mainly reported being unconcerned about their conception. The fact that none of the adolescents conceived through any of the types of reproductive donation were found to feel distressed about their conception is of considerable importance given such longstanding concerns. The findings also indicate that it is important to differentiate between adolescents’ feelings about their conception, and their feelings about the surrogate or donor. How these feelings may change as the adolescents enter adulthood remains to be seen.
